# The Involvement of Glycerophospholipids in Susceptibility of Maize to Gibberella Root Rot Revealed by Comparative Metabolomics and Mass Spectrometry Imaging Joint Analysis

**DOI:** 10.3390/plants14091376

**Published:** 2025-05-01

**Authors:** Qing Wang, Zi’an Zhao, Xin Li, Xiquan Gao

**Affiliations:** 1 State Key Laboratory for Crop Genetics & Germplasm Enhancement and Utilization, Nanjing Agricultural University, Nanjing 210095, China; qingwang@njau.edu.cn (Q.W.); 2022101143@stu.njau.edu.cn (Z.Z.); 2020101078@stu.njau.edu.cn (X.L.); 2 College of Agriculture, Nanjing Agricultural University, Nanjing 210095, China; 3 Jiangsu Collaborative Innovation Center for Modern Crop Production, Nanjing Agricultural University, Nanjing 210095, China

**Keywords:** *Zea mays*, Gibberella root rot, lysophosphatidylethanolamine, lysophosphatidylcholine, phospholipase, lysophospholipases, mass spectrometry imaging

## Abstract

Gibberella root rot (GRR), caused by *Fusarium graminearum*, is one of the major threats to maize production. However, the mechanism underlying maize’s response to GRR is not fully understood. Multi-omics study incorporating metabolomics reveals insights into maize–pathogen interactions. Using metabolomics and mass spectrometry imaging (MSI), maize inbred lines with GRR resistance (W438) and susceptibility (335M) were deployed to characterize specific metabolites associated with GRR. Analysis of significantly altered metabolites suggested that glycerophospholipid metabolism was highly associated with GRR resistance or susceptibility. Furthermore, the distinct accumulation of lysophosphatidylethanolamine (lysoPE) and lysophosphatidylcholine (lysoPC) from glycerophospholipid metabolism, along with the significant up-regulation of phospholipase (*PLA*) gene in the susceptible line, suggested that high levels of lysoPC and lysoPE contributed to GRR susceptibility. Meanwhile, genes encoding lysophospholipase (LPLA), the detoxification enzymes of lysoPC, were significantly activated in both genotypes. However, the significantly higher expression of *LPLAs* in the resistant line corresponded to a significant increase in the content of non-toxic sn-glycero-3-phosphocholine, whereas this increase was not observed in the susceptible line. MSI analysis revealed the involvement of other potential phospholipids in GRR susceptibility. Taken together, maintaining an appropriate concentration of lysophospholipids is crucial for their role in the signaling pathway that triggers GRR resistance without causing damage to maize roots.

## 1. Introduction

Maize (*Zea mays* L.) is one of the major crop species worldwide. In China, over 29,000 million tons of maize were produced in 2024. However, approximately more than 10% of yield loss was estimated due to various diseases [1]. *Fusarium graminearum* Schwabe (teleomorph *Gibberella zeae* (Schwein) Petch) (*F. graminearum*) is one of the major devastating fungal pathogens in wheat–maize-producing area. Along with *F. pseudograminearum*, *F. culmorum*, *F. avenaceum*, are the predominant pathogens of crown rot in wheat (*Triticum aestivum*) in the North China Plain [2,3]. Additionally, soil-borne pathogens such as *Bipolaris sorokiniana* and *Rhizoctonia cerealis*, known to cause common root rot and sharp eyespot (sheath blight) in wheat, have been reported to infect maize, barley (*Hordeum vulgare*), rice (*Oryza sativa*), and many other grass species [3,4,5].

As a soil-borne pathogen, *F. graminearum* can overwinter as sporangia and hyphae on crop residues and infect the roots of germinating maize and wheat seedlings under favorable temperature and humidity conditions. *F. graminearum* can cause maize ear rot, stalk rot, and root/crown rot, so-called Gibberella ear rot (GER), Gibberella stalk rot (GSR), and Gibberella root rot (GRR), respectively. GRR afflicts considerable yield reduction annually in North America [6], usually resulting in abnormal growth or even death at seedling stage. Over the past few years, root rot of wheat caused by *Fusarium* species has spread rapidly and become more severe [2,7], largely due to the high humidity and temperature. Therefore, it is foreseeable that GRR could be next the epidemic disease in maize, especially in areas using a maize–wheat rotation cropping system. In recent years, research on resistance to GSR and GER have been greatly advanced. However, the understanding of maize resistance to GRR is very limited.

Metabolites have been widely known to play important roles in maize resistance to diverse pathogens. A previous study reported that near-isogenic lines (NIL) carrying a GSR-resistant *qRfg1* allele or gene exhibited significant lower fungal biomass or disease severity index than susceptible-NIL (S-NIL) upon infection with *F. graminearum* [8,9]. Prior to infection, lignin and phenolic acids associated with fungal resistance were found to be significantly accumulated in R-NIL; however, the differences were no longer evident between R-NIL and S-NIL after infection [8]. Antifungal metabolites smiglaside C and smilaside A were discovered through comparative metabolomics conducted using GRR-resistant and susceptible inbred lines. Furthermore, based on the varying ratio of smilaside A to smiglaside C in recombinant inbred lines (RIL), an integrated study combining mQTL mapping and comparative transcriptomics revealed that ethylene signaling gene *ZmEIN2* negatively regulates smilaside A/smiglaside C ratio, which in turn affects GRR resistance to *F. graminearum* [10]. Thus, the identification of fungal-induced specialized metabolites provides a promising strategy for studying host–fungal interaction. For instance, comparative metabolomics identified fungus-elicited O-methylflavonoids, such as xilonenin, which significantly inhibit the growth of *F. graminearum* and *F. verticillioides* [11]. Using RIL population and xilonenin levels, association mapping identified flavonoid O-methyltransferase 2 (FOMT2), which was confirmed to catalyze the formation of xilonenin [11]. Clearly, mining resistance-related genes by recognizing specific metabolites associated with resistance or susceptibility is an effective approach to elucidate the mechanism of maize in response to *F. graminearum*.

In addition to classical metabolomics, as a complement, the visualized metabolites detection approach has been utilized in plant–pathogen interaction research. Mass spectrometry imaging (MSI) is a method of scanning sections of a sample and generating images of the intensity distribution of analyte ions [12]. MSI provides spatial and molecular information for a disease-related metabolite at tissue- and single-cell level [13]. MSI has been utilized to map defense-related compound in disease-infected plants in previous studies. For example, using LDI-MSI technology, banana-specific phytoalexins and phenylphenalenones were identified and shown to improve resistance to *Radopholus similis* [14]. MSI has also identified mycotoxin and antifungal metabolites induced by Fusarium root rot in wheat [13], oxylipins associated with resistance to Fusarium head blight in wheat seeds [15], and anthocyanin and chlorophyll metabolism induced by aflatoxin B1 (AFB1) treatment in maize roots, along with antimicrobial or antioxidant effects in maize stem bases [16]. Furthermore, metabolites of serotonin and melanin pathway have been identified as pathogenicity factors of *Magnaporthe oryzae* infecting barley [17]. These findings underscore the significance of MSI in elucidating the complex interactions between plants and pathogens.

Phospholipids, including phosphatidic acid (PA), phosphatidylcholine (PC), phosphatidylethanolamine (PE), and phosphatidylinositols (PIs), are the main components of plant cell membranes. These lipids have been extensively reported as signaling compounds involved in plant–pathogen interaction [18,19,20,21]. Among them, PE and PC can be hydrolyzed by Phospholipase A1 (PLA1) and Phospholipase A2 (PLA2) at the sn-1 and sn-2 positions, respectively, to generate lysophosphatidylethanolamine (lysoPE) and lysophosphatidylcholine (lysoPC). Regarding the function of PLAs, lysoPE, and lysoPC in plants upon pathogen infection, conflicting conclusions have been reported. For instance, after inoculation with *Phytophthora parasitica var. nicotianae* (Ppn), susceptible tobacco (*N. tabacum* L. cv Wisconsin 38) exhibited significant up-regulation of *Nt1PLA2* and *Nt2PLA2* [21], accompanied by high reactive oxygen species (ROS) accumulation and cell damage [22]. Moreover, exogenous application of lysoPC (C18:1(9Z)) not only led to ROS accumulation and accelerated cell death but also induced the expression of pathogenesis-related (PR) genes [21]. Therefore, pathogen-induced lysoPC seemed to be associated with host susceptibility to pathogens. In line with this result, in an abiotic stress study, lysoPC and lysoPE levels significantly increased in a cold-sensitive tomato line compared to a tolerance genotype, which displayed stronger ROS scavenging capacities [23]. However, contradictory results indicated that lysoPE induced resistance. In Arabidopsis, mutant of lysoPE acyltransferase *lpeat1* and *lpeat2* showed increased lysoPE in situ, whereas the PLA2 mutant *pla2-alpha* manifested lysoPE deficiency. Upon infection with *Botrytis cinerea*, *lpeat1* and *lpeat2* showed increased resistance, whereas *pla2-alpha* was more susceptible [20]. Additionally, lysoPE has been reported to enhance plant resistance against necrotrophic and hemibiotrophic pathogens, likely via activating the jasmonic acid (JA) and salicylic acid (SA) signaling pathways [20,24]. Following inoculation with *Plasmodiophora brassicae*, three *PLA1* mutants and one patatin-like PLA (*pPLA*) mutant displayed increased susceptibility, suggesting that PLA1 and pPLA might be involved in the resistance of *Arabidopsis thaliana* to Clubroot [25]. In cotton, lysophospholipase GhLPL2 was found to interact with necrosis/ethylene-inducing peptide 1 (Nep1)-like proteins (NLP) from *Verticillium dahlia* to suppress the cytotoxicity of VdNLP1. Surprisingly, *GhLPL2* silencing via virus-induced gene silencing (VIGS) in cotton and overexpressing *GhLPL2* in Arabidopsis and cotton all displayed unexpectedly increased resistance to *V. dahlia* [26]. Despite the above, the function of the lysophospholipids, phospholipases, and lysophospholipases (LPLAs) in maize responsive to pathogen infection has not been fully investigated.

To gain insights into the role of secondary metabolites involved in maize response to GRR disease, comparative metabolomics was deployed using a pair of maize inbred lines W438 and 335M with contrast GRR resistance level. In addition, expression levels of genes encoding key enzymes in glycerophospholipid pathway were investigated and specific phospholipid compound was characterized using MSI. The results showed that lysoPC, lysoPE, and PLAs likely contribute to the GRR susceptibility in maize, which could be utilized as potential target for maize breeding to improve GRR resistance through genome editing.

## 2. Results

### 2.1. Morphological and Pathological Differences Between Maize Inbred Lines to GRR

**Figure 1:**
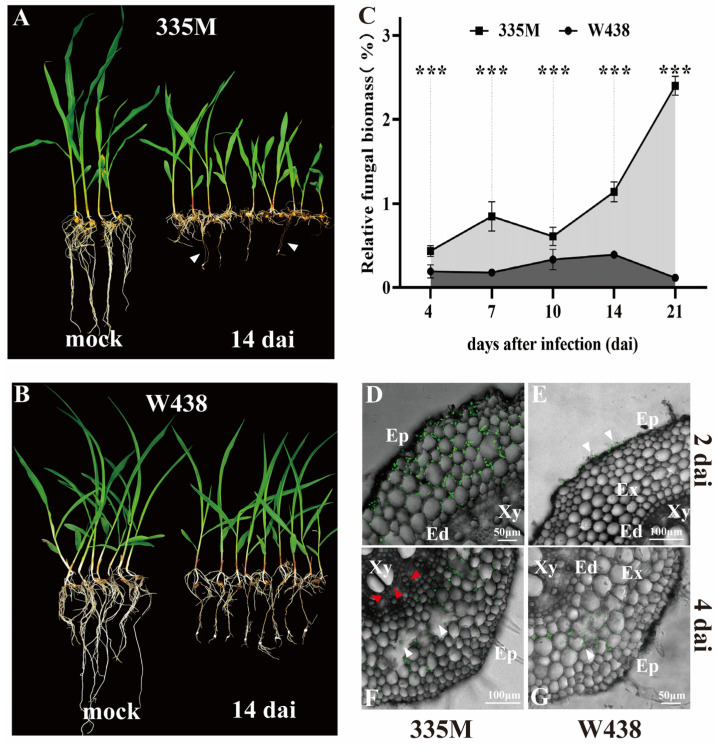
GRR phenotypes of W438 (resistant) and 335M (susceptible) upon *F. graminearum* infection. (**A**) Growth suppression of root and shoot was observed in 335M at 14 dai. The white arrows indicate severe necrosis on roots. (**B**) W438 displayed fewer lesion on the root and minimal effect on the shoot growth at 14 dpi. (**C**) Relative fungal biomass measured by qPCR at 4, 7, 10, 14, and 21 dai. The data are presented as mean ± SD (n = 10), and asterisks indicate statistical significance by *t*-test (***, *p* ≤ 0.001). (**D**,**E**) Root cross-sections of 335M and W438 at 2 dai. Green signal indicating the position of fungal hyphae within root tissue. (**F**) Fungal hyphae colonization in 335M at 4 dai. Red arrow indicates the spreading of fungus inside xylem tissue, with the cavity (white arrow) observed within exodermis tissue. (**G**) Fungal hyphae colonization in W438 at 4dai. The fungus was confined to epidermis and exodermis. Image D to G were obtained using confocal laser scanning microscopy on roots which was stained with WGA Alexa Fluor 488 (green signals). C: cavity; Ed: endodermis; Ep: epidermis; Ex: exodermis; Xy: xylem.

Previous research has indicated that resistance to GSR might be relevant to GRR resistance [8,9]. Therefore, a pair of lines showing contrast GSR phenotype (resistant line W438 and susceptible line 335M) were examined for GRR response. Three independent biological replicates supported similar GRR levels as that of GSR of two lines examined. Specifically, severe necrosis was observed on the root of 335M at 4 days after infection (dai) (Figure S1A, white arrow), while no obvious symptoms were found on W438 (Figure S1B). By 14 dai, *F. graminearum*-induced necrosis inhibited the elongation of the primary root and shoot growth in 335M (Figure 1A, white arrow). In contrast, W438 displayed lighter symptoms on primary root, yet a more extensive fibrous root system (Figure 1B). Quantitative PCR (qPCR) analysis demonstrated significantly higher relative fungal biomass in 335M compared to W438 across all time points examined (Figure 1C). In three independent biological replicates, 335M showed progressive fungal proliferation, with relative fungal biomass increasing from 0.431% at 4 dai to 2.44% by 21 dai. In contrast, W438 maintained consistently lower colonization levels, ranging from 0.189% at 4 dai to 0.392% at 14 dai, followed by a decline to 0.14% at 21 dai (Supplementary Table S1). Consistent with the differences in plant growth and fugal biomass accumulation, histological staining results showed that *F. graminearum* hyphae penetrated the epidermis, colonized exodermis and reached endodermis cells in 335M as early as 2 dai (Figure 1D, green signal). However, fungal hyphae were still confined to the vicinity of epidermis cells in W438 at 2 dai (Figure 1E, white arrow). At 4 dai, hyphae in 335M had breached the endodermis and were observed near the xylem tissue (Figure 1F, red arrow); in particular, some cavity (white arrow) was presented in cortical cells, indicating the decay of root tissue. Meanwhile, although *F. graminearum* had possessed the partial exodermis in W438 root (Figure 1G), it was only observed within brown lesions. These findings suggested that inbred line 335M exhibited GRR susceptibility characterized by high fungal biomass and faster infection process, whereas W438 performed oppositely.

### 2.2. Metabolomic Changes Between GRR-Resistant and Susceptible Lines upon F. graminearum Infection

To investigate whether the metabolomic changes are associated with GRR resistance or susceptibility, widely targeted comparative metabolomic analysis was performed using the roots and basal part of the stem from W438 and 335M seedlings at 4 dai, both with and without *F. graminearum* inoculation. In total, more than one thousand metabolites were detected, including Alkaloids, Amino acids and derivatives, Flavonoids, Lignans and Coumarins, Lipids, Nucleotides and derivatives, Organic acids, Others and Phenolic acids. Principal Component Analysis (PCA) analysis revealed overall distinct metabolomic difference between W438 and 335M. Upon *F. graminearum* infection, about 70% of the variance was explained between W438 and 335M compared to their pre-inoculation states, with good reproducibility among samples within each group (Figure S2A). In W438, a total of 195 metabolites were up-regulated, while 17 metabolites were down-regulated. The number of differentially expressed metabolites (DEMs) increased in 335M, with 281 metabolites up-regulated and 49 down-regulated (Figure S2B).

**Figure 2:**
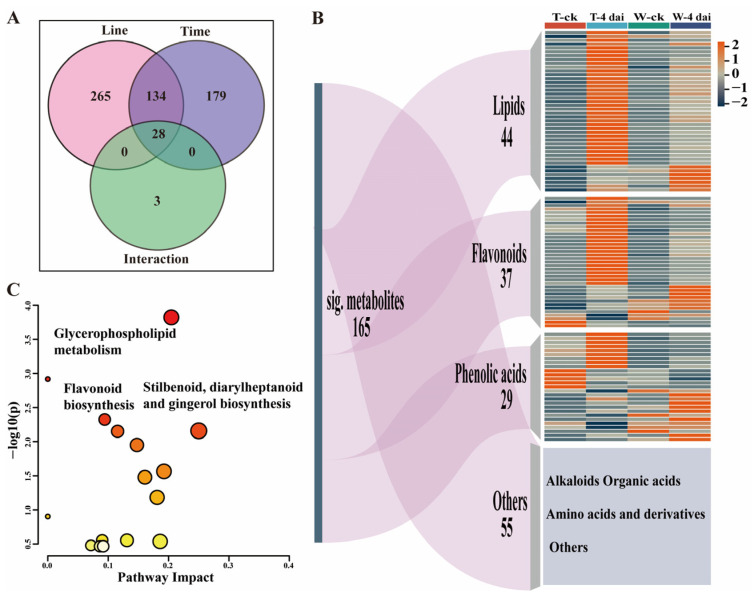
Comparative metabolomics analysis in W438 and 335M following *F. graminearum* inoculation. (**A**) Venn diagram illustrating the number of differentially expressed metabolites (DEMs) in W438 and 335M at 4 dai. (**B**) A combination of Sankey diagram and heatmap depicting the distribution of metabolite types and the expression patterns of DEMs. The color bar on the right represents the fold change in metabolite contents. T: 335M; W: W438. (**C**) Enrichment analysis of metabolomic pathways highly associated with the DEMs between W438 and 335M. The bubbles represent different pathways, with their size proportional to the number of metabolites belonging to each pathway, and dark colors indicate a high possibility of the pathway associated with the GRR phenotypes.

To further identify the significant changes in metabolites between 335M and W438 after *F. graminearum* inoculation, a two-way repeated ANOVA was conducted. In total, 165 metabolites exhibited distinct pattern of changes between two genotypes at 4 dai (Figure 2A). Among these significant changed compounds, 44 were lipids, 37 were flavonoid, 29 were phenolic acids, and 55 belonged to categories including alkaloids organic acids, amino acids, and derivatives (Figure 2B). Pathway impact analysis suggested that glycerophospholipid metabolism; flavonoid biosynthesis; and stilbenoid, diarylheptanoid and gingerol biosynthesis contributed to the significant alterations in metabolites (Figure 2C), suggesting that these metabolic pathways are most likely associated with the different GRR response between W438 and 335M.

Among the 37 significantly altered flavonoids, only 15 could be annotated in the KEGG database. These were categorized into flavone and flavonol, isoflavonoid, and flavonoid pathways. This dispersed distribution prevented definitive conclusions regarding which specific pathway might be associated with GRR resistance or susceptibility. In contrast, 35 significantly altered lysophospholipids out of 44 lipids clustered specifically in the glycerophospholipid pathway, suggesting this pathway may represent a more promising research focus for further investigation of GRR resistance or susceptibility mechanisms.

### 2.3. Differential Accumulation of Lysophospholipids and Expression of Related Genes

**Figure 3:**
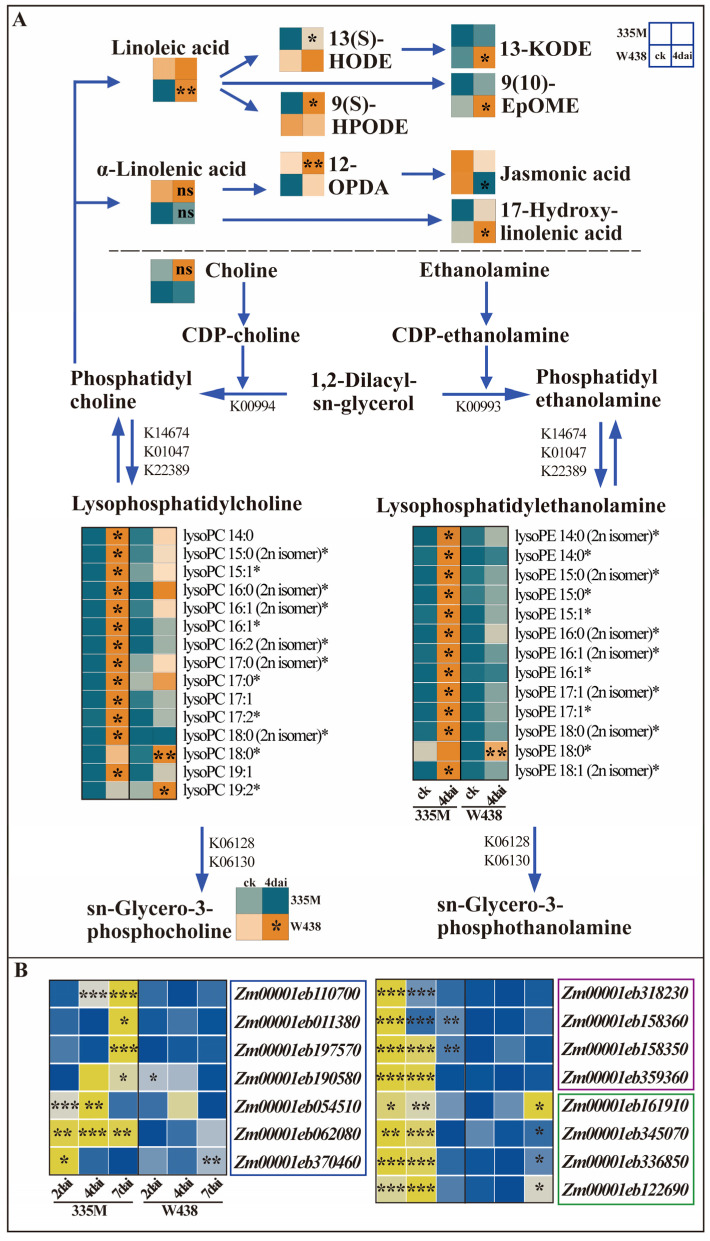
Dynamic changes in metabolite contents and expression levels of corresponding genes in glycerophospholipid, linoleic acid, and α-linolenic acid pathways in W438 and 335M at 4 dai. (**A**) Comparison of lysoPCs, lysoPEs, and oxylipin contents between W438 and 335M at 4 dai. K numbers represent enzyme code in KEGG. Asterisks indicate statistical significance (* *p* ≤ 0.05; ** *p* ≤ 0.01; two-way repeated ANOVA). (**B**) Heatmap of gene expression levels quantified by qRT-PCR for *PLA1*, *PLA2*, and *LPLA* in W438 and 335M at 2, 4, and 7 dai, respectively. *PLA2* genes shown in blue squares, *PLA1* genes in purple squares, and *LPLA* in green squares. The asterisks indicate statistical significance (* *p* ≤ 0.05; ** *p* ≤ 0.01; *** *p* ≤ 0.001, ns, not significant, two-way repeated ANOVA).

To gain more insights into the roles of glycerophospholipid metabolism in maize’s response to GRR, a detailed analysis was performed on each significantly altered lipids. Among the 44 significantly accumulated lipids, 35 were lysophospholipids comprising nineteen lysoPCs and sixteen lysoPEs. In 335M, thirteen out of the fifteen detected lysoPCs exhibited significantly increased levels, with the exceptions of lysoPC 18:0 and lysoPC 19:2, which were more abundant in W438 (Figure 3A). Similarly, twelve out of the thirteen lysoPEs were significantly elevated in 335M, except that for lysoPE 18:0 (Figure 3A). Consistent with previous studies reporting increased lysoPCs (C18:1, C18:2, and C18:3) upon pathogen inoculation, the concentration of lysoPC/lysoPE (C 18:1, C 18:2, and C 18:3) in this research also increased in both genotypes. However, there was no significant differences between both genotypes (Figure S3A). Interestingly, lysoPC and lysoPE containing long-chain fatty acids (20:2, 20:4, 22:4, and 22:5) were also significantly accumulated in 335M (Figure S3B). Given the lack of evidence for endogenous long-chain fatty acid synthesis in maize roots, these lysoPCs and lysoPEs likely originate from *F. graminearum*, aligning with the higher fungal biomass detected in 335M (Figure 1C). Although multiple lysoPCs and lysoPEs were detected, their precursors, PC and PE, were not identified. Choline, a substrate of PC synthesis, was detected no difference between 335M and W438. Additionally, sn-Glycero-3-phosphocholine, a product of lysoPC hydrolysis, was detected to be significantly accumulated in W438 upon fungal infection (Figure 3A).

Beyond generating lysoPCs, PC hydrolysis also liberates linoleic acid and α-linolenic acid. In the α-linolenic acid pathway, (9S, 13S, 15Z)-12-Oxophyto-10, 15-dienoate (12-OPDA) accumulated significantly in 335M, whereas the content of jasmonic acid (JA) decreased in W438, corroborating our earlier findings [27]. In the linoleic acid pathway, linoleic acid, (9Z, 11E)-13-Oxooctadeca-9,11-dienoic acid (13-KODE), and (9R,10S)-(12Z)-9,10-Epoxyoctadecenoic acid (9 (10)-EpOME) accumulated significantly in W438, while (13S)-Hydroxyoctadecadienoic acid (13 (S)-HODE) and (9S,10E,12Z)-9-Hydroperoxy-10,12-octadecadienoate (9 (S)-HPODE) were elevated in 335M.

To understand whether these catalytic enzymes are involved in the metabolism of lysoPC and lysoPE at the transcriptional level, the expression of genes related to the production and hydrolysis of lysoPC and lysoPE were analyzed using qRT-PCR. PLA1 and PLA2 are the two main enzymes responsible for the synthesis of lysoPC and lysoPE. Therefore, the expression of ten *PLA2* (K01047, K14674) homolog genes and nine *PLA1* (K22389) homolog genes were quantified in both lines at different time points upon infection with *F. graminearum*. Among these genes, seven *PLA2* and four *PLA1* genes were significantly up-regulated in 335M at 2, 4, and 7 dai, but no distinct changes were found in W438 (Figure 3B, blue and purple square). The expression patterns of *PLA1* and *PLA2* were consistent with the changes in lysoPC and lysoPE contents in 335M and W438, respectively. Furthermore, the expression of genes encoding lysophospholipase (LPLA), which hydrolyze lysoPC and lysoPE to generate glycerophosphodiesters, was also measured. Four out of five *LPLA* (K06128, K06130) homolog genes were significantly up-regulated in 335M at earlier time points (2 and 4 dai), whereas these four genes were significantly induced at a later time point (7 dai) in W438 (Figure 3B, green square). The expression of *LPLAs* supported the distinct accumulation of sn-Glycero-3-phosphocholine in W438 but did not correspond to the non-significant change observed in 335M. Taken together, the metabolites and gene expression profiling suggested that lysophospholipids, especially lysoPCs and lysoPEs, might contribute to GRR susceptibility.

### 2.4. Temporal and Spatial Distribution of GRR Induced Metabolites

**Figure 4:**
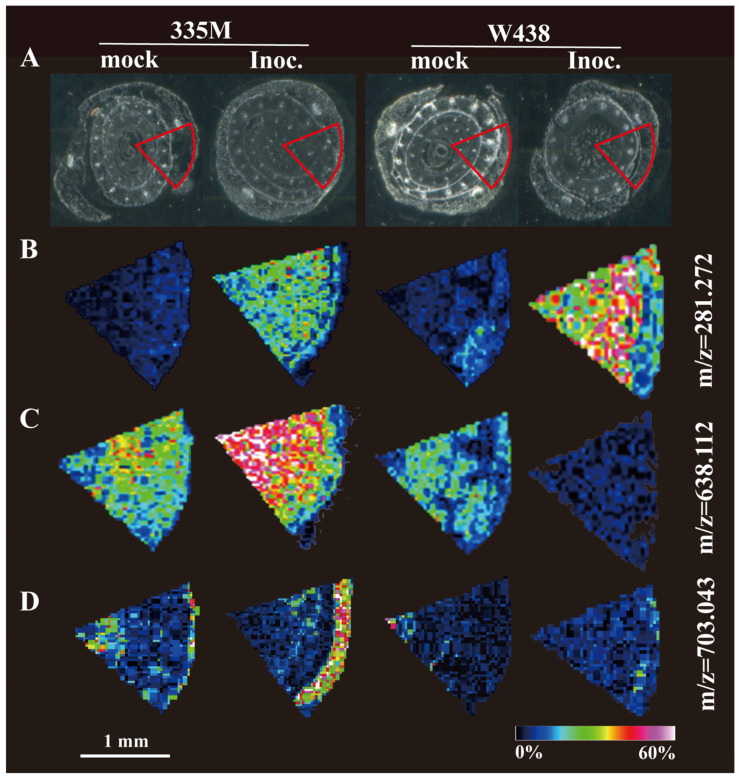
Comparative MALDI-MSI analysis of stem base sections from GRR-infected and non-infected maize seedlings. (**A**) Optical images of *F. graminearum*-infected and non-infected stem base section from W438 and 335M at 4 dai. The data display area is represented by the red fan-shaped sector. (**B**–**D**) MSI exhibited the distribution of putative compounds within stem base tissue of W438 and 335M at 4 dai. Color scales indicate the intensity of the extracted ion, normalized to total ion current.

Mass spectrometry imaging (MSI) enables the in situ detection of metabolites within the tissues. To characterize the metabolites that might be associated with GRR phenotype, preliminary MSI analysis was performed using the infected and non-infected stem base sections of W438 and 335M (Figure 4A). It clearly showed that the differential metabolites vary in either the levels or the tissue-specific patterns between two genotypes. For example, putatively identified N(6)-Methyladenosine (m/z 281.272) was widely mapped to entire tissue section in both genotypes following *F. graminearum* infection, with the high intensity of ion indicating the high content of this compound in W438 at 4 dai (Figure 4B). Presumed to be a diglucuronidated luteolin derivative, luteolin-7-O-glucuronide-(2→1)-glucuronide (m/z 638.112) was found to accumulate exclusively in 335M at 4 dai, whereas its content in pathogen-infected W438 decreased when compared to an uninfected tissue section (Figure 4C). Interestingly, speculatively identified as a type of sphingomyelin, N-palmitoyl-D-erythro-sphingosylphosphorylcholine (16:0 SM (d18:1/16:0), m/z 703.043) seemed to accumulate in vascular bundle of 335M prior to infection, whereas it was specifically accumulated in the leaf sheath of 335M upon infection. However, a very low amount of this compound was visualized in W438 at 4 dai (Figure 4D).

## 3. Discussion

The function of lysoPCs, lysoPEs, and their associated enzymes in plant–pathogen interaction have been extensively investigated, primarily focusing on their direct or indirect involvement in pathogen-triggered signaling pathway. It has been proposed that phospholipases and lysophospholipids play positive roles in plant disease resistance. For example, exogenous lysoPE increases resistance against hemibiotrophic and necrotrophic pathogens in *Arabidopsis thaliana* by triggering SA or JA signaling [20,24]. LysoPC 17:1 was identified from wild potato species *Solanum bulbocastanum* that showed resistance to *Phytophthora infestans* via inhibiting its spore germination and mycelial growth [28]. Patatin-like phospholipase *CaPLP1* from pepper (*Capsicum annuum*) was found to be involved in defense against *Xanthomonas campestris pv. vesicatoria* (*Xcv*). *CaPLP1*-silenced mutant showed increased susceptibility to Xcv, and overexpression of *CaPLP1* in *Arabidopsis* enhanced resistance to *Pseudomonas syringae pv. tomato* (Pst) and *Hyaloperonospora arabidopsidis* infection [29]. Moreover, *GhPLP2*-silenced cotton showed increase susceptibility to *Verticillium dahlia*, while overexpression of *GhPLP2* in *Arabidopsis* enhanced the resistance to *V. dahlia* [30]. Meanwhile, *PLP2* from *Arabidopsis* contributed to resistance against *Cucumber mosaic virus* [31].

Despite of the fact that phospholipases and lysophospholipids could play roles in plant disease resistance, it needs to be clarified that the expression of phospholipases and accumulation of lysophospholipids were often associated with cell death or reactive oxygen species (ROS). For instance, lysoPE treatment led to increased hydrogen peroxide (H_2_O_2_) levels accompanied by strong activation of H_2_O_2_ inducible genes [20,24]. ROS level and hypersensitive cell death were weakened in the *CaPLP1*-silenced pepper plants, while *CaPLP1* overexpression leaves showed enhanced ROS burst [29]. *GhPLP2*-silenced cotton plants also exhibited reduced hypersensitive response, callose deposition, and H_2_O_2_ accumulation induced by *Verticillium dahlia* elicitor [30]. Interestingly, *AtPLP2* genetically contributed to cell death and induced accumulation of tissue damage related compounds [32]. The fact that the cell death and ROS production resulted from a lysophospholipid-dependent pathway could partially support our findings. The increased lysoPC and lysoPE levels, resulting from the significantly higher expression level of *ZmPLA*s in 335M (Figure 3), may have triggered an ROS burst. Furthermore, LysoPC and lysoPE could act as toxic compounds, leading to more severe necrosis and thereby beneficiating the invasion process of *F. graminearum* (Figure 1), ultimately resulting in the GRR susceptibility in 335M. In accordance with our result, pathogen-induced lysoPC enhanced the susceptibility accompanied by ROS biosynthesis, while lysoPC 18:1 treatment resulted in cell death in tobacco [21]. The increased amount of pathogen-induced lysoPC (C16:0 (1), C18:2 (4), and C18:3 (5)) at 48 h together with high level of ROS accumulation are in line with pathogen growth and host cell damage [22,33]. Therefore, one possibility cannot be excluded: the highly accumulated lysophospholipids in susceptible line 335M are generated by *F. graminearum*, which is in line with its higher level of susceptibility. The finding that glycerophospholipids are associated with GRR susceptibility provides an alternative strategy to improve maize resistance to GRR through knocking down expression levels of genes encoding specific glycerophospholipids by CRISPR–Cas-based genome editing.

To avoid the damage caused by excessive lysophospholipids, *Eschscholzia californica* possessed a high level of reacylation to down-regulate the level of lysoPC, which is a detoxification reaction to protect plants from the toxic lysoPC [34]. Overexpression of *LPLA* increased the hydrolysis to lysoPC, which in turn induced a higher level of tolerance to cadmium-induced oxidative stress in *Arabidopsis* [35]. Thus, the utilization of the antipathogenic potential of lysoPC largely depends on achieving an appropriate concentration to avoid toxic effects [21,36]. In this study, four hydrolyzation enzyme genes of lysoPC and lysoPE were significantly up-regulated at 7 dai in W438, which might explain why non-toxic sn-Glycero-3-phosphocholine was significantly accumulated in W438 (Figure 3). In particular, the high level of sn-Glycero-3-phosphocholine could explain the low concentration of lysoPC accompanied by less necrosis on the root of resistant line W438. It is puzzling that the significant up-regulation in 335M at 2 and 4 dai was not consistent with the non-significant change in sn-Glycero-3-phosphocholine, warranting further investigation.

Although eight significantly differential oxylipins were detected in the α-linolenic acid and linoleic acid pathways, their distribution across five distinct sub-pathways, with divergent accumulation patterns, were found between upstream and downstream products in W438 and 335M. These findings preclude definitive conclusions. Notably, the accumulation patterns of 9, 10-EpOME and JA in W438 (Figure 3A) align with our previous studies. JA levels were significantly elevated in the GSR-susceptible mutant *zmlox5-3* compared to the GSR-resistant line W438. In contrast, 9-oxylipins (including 9, 10-EpOME) were significantly induced in W438 but unchanged in the susceptible mutant, suggesting a positive correlation between high JA levels and enhanced GSR susceptibility, and the contribution of 9-oxylipins to GSR resistance [27].

Directly mapping metabolites within disease-relevant tissues offers a promising strategy for identifying the pathways and/or specific metabolites involved in plant–pathogen interactions. In this study, changes in the levels of N(6)-Methyladenosine (m^6^A) following infection by *F. graminearum* were detected. As the most prevalent internal modification in eukaryotic mRNA, m^6^A has been previously reported to enhance powdery mildew resistance in apples when its recognition protein is overexpressed [37]. Additionally, m^6^A demethylases of *Arabidopsis* have been shown to negatively regulate virus infection by removing m^6^A from virus RNA [38]. Thus, the finding that m^6^A content was strongly increased in GRR-resistant line W438 indicated that m^6^A might play a role in modulating GRR resistance.

Luteolin displays a broad spectrum of biological activities, functioning as a cytotoxic agent, anti-inflammatory compound, antioxidant, and antimicrobial substance [39,40]. For example, *Lippia alba* leaf extract containing luteolin-7-O-glucuronide exhibited insecticidal efficacy against fall armyworm (*Spodoptera frugiperda*) in maize [41]. The increase in luteolin derivatives and associated flavonoids have been documented in root responses to *Funneliformis mosseae* colonization in wheat [42], as well as to *Colletotrichum graminicola* infection and soil salinization in maize [43,44]. However, luteolin derivatives can be also negatively related to stress response, as the content of luteolin 7-O-glucuronide significantly declined in carrot resistance line against *Alternaria dauci* [45] and similarly decreased in *Rhododendron chrysanthum* Pall. under UV-B stress [46]. This context suggests that the observed increase in luteolin-7-O-glucuronide in 335M (Figure 4C), contrasted with its reduction in resistant W438, may inversely associate with resistance to GRR resistance.

Leveraging the benefit of MSI to mapping compounds in situ, one of sphingomyelin, 16:0 SM (d18:1/16:0) was strongly induced in 335M tissues, particularly accumulated in leaf sheath upon fungal infection (Figure 4D). This accumulation is likely associated with the infection process as it spreads from the root to the base of the stem. As a major component of cell membranes, sphingomyelin is composed of ceramide and phosphatidylcholine. Studies on plants have predominantly centered on sphingomyelin’s role in intra- and inter-membrane transport [47,48]. Additionally, its capacity to bind cholesterol and form lipid rafts is another significant characteristic of sphingomyelin. This complex involves the maintenance of membrane homeostasis, cellular proliferation, and apoptosis. Notably, sphingomyelin is one of the sources of ceramide [49]. Ceramides have been found to induce the hypersensitive response (HR) or programmed cell death (PCD) in plants [50] and to modulate resistance against pathogens [51,52]. Therefore, the specific accumulation of 16:0 SM in the leaf sheath might be relevant to membrane repairing and the activation of signaling pathway responding to GRR.

In the context of plant–pathogen interaction research, a single time point measurement of MSI is insufficient to capture the dynamic changes of metabolites in response to GRR. Consequently, a strategic and cost-effective approach is to identify disease-related metabolites firstly through classical untargeted metabolomics. Subsequently, conducting targeted MSI of these metabolites at three or more time points will yield more compelling results. In the future, a more complex and dynamic study of MSI on targeted metabolites through entire interaction process between plant and pathogen will enable a deeper understanding of the temporal and spatial dynamics of metabolites in response to GRR.

## 4. Materials and Methods

### 4.1. Plant Materials and Growth

Maize inbred lines W438 and 335M are propagated and maintained in Gao lab. Prior to sowing, maize seeds were sterilized in 5% sodium hypochlorite solution (tap water/20% NaClO, vol/vol) for 5 min, and washed ten times with tap water. Seeds were soaked overnight in sterilized water and then transferred to wet filter paper in Petri dish, keeping wet for 3 days, until most of seeds germinated. The germinated seeds were carefully sowed in autoclaved sand (construction yellow sand) and cultivated in climate chamber with a 14 h cool-white light illumination at 22–25 °C and 60% humidity until seedlings reach to the stage of V3 [53].

### 4.2. Fungal Strain and Preparation of Inoculum

*F. graminearum* strain PH-1 was cultured on potato dextrose agar medium under conditions identical to those for maize seedlings for one week, until the Petri dish was covered with *F. graminearum* mycelium. Ground corn kernels were immersed in water for 1 h, and the excess water was drained off. Approximately 400 mL of soaked ground corn kernels was added to 1L Erlenmeyer flask. The flasks containing ground corn kernels were covered with four lays of newspaper, and autoclaved for 20 min at 121 °C. After cooling, 20 pieces of *F. graminearum* agar plugs, each with a diameter of 0.5 cm, were added into the flask and thoroughly mixed with the autoclaved ground corn kernels. The mixture was then cultured for 5–7 days under conditions identical to those for maize seedlings, until the mycelium was distributed throughout the ground corn kernels.

### 4.3. Kernel–Sand Mixture Inoculation and Sampling

Before infection, maize seedlings were carefully removed from original pots. Five maize seedlings were transferred into one pot, which is filled with the mixture of 10 g of inoculated ground corn kernels and autoclaved sand. For mock, every five seedlings were planted with autoclaved sand alone. All seedlings were cultivated in climate chamber with a 14 h cool-white light illumination at 22–25 °C and 60% humidity. For qPCR-based relative fungal biomass measurement, root samples were collected at 4, 7, 10, 14, and 21 dai. At each time point, roots of five seedlings were collected to form one sample with two replicates per sample. For microscopy observation, the entire plant was harvested at 2, 4, 7, 10, and 14 dai, with ten infected seedling and five mock seedlings per genotype at per time point. For metabolomics analysis, 20 infected seedling roots and 20 mock roots were collected per genotype at 4 dai. For gene expression analysis, root samples were collected at 2, 4, and 7 dai, with ten infected seedling roots and ten uninfected seedling roots were harvested as one sample per genotype.

### 4.4. DNA Isolation from Maize Roots and Fungal Mycelia

The extraction of maize or *F. graminearum* genomic DNA (gDNA) was performed using a modified CTAB method [54]. The gDNA of uninfected seedling leaves was serially diluted with sterile double-distilled water (100, 50, 25, 6.25, 1.5625, and 0.39 ng/μL) as external maize DNA standard. *F. graminearum* mycelium was harvested from PDA medium and ground in liquid nitrogen. The gDNA of mycelium was serially diluted with sterile double-distilled water (50, 10, 5, 1, 0.5, and 0.1 ng/μL) as external fungal DNA standard. The gDNA of inoculated seedling roots, which was used to quantify root and *F. graminearum* biomass, was diluted to 50 ng/μL before use. gDNA concentration was determined using the NanoDrop N-1000 (Thermo Fisher Scientific, Waltham, MA, USA).

### 4.5. qPCR Measurement of Fungal and Root gDNA

To quantify the relative biomass of *F. graminearum*, absolute quantification analyses were performed on a Roche LightCycler^®^ 480 real-time PCR system (Hoffmann-La Roche Ltd., Basel, Switzerland). Primers of *F. graminearum* and maize Tubulin gene were used to quantify fungal and root DNA; both primers was tested for expression stability and specificity. Information on the primers is listed in Table S2. The qPCR amplification mix consisted of 1 μL of template DNA, 5 μL of F488 SYBR mixture (BestEnzymes Biotech Co., Ltd., Lianyungang, China), 2 μL of double-distilled water, and 1 μL each of forward and reverse primer (10 pmol/μL). qRT-PCR was performed in accordance with modified protocol [54]. A higher relative fungal biomass value indicates greater infection severity and higher host susceptibility. The relative fungal biomass was calculated based on the modified published method [55]:

$$relative\;fungal\;biomass\;\%=\frac{Fusarium\;DNA\;(ng)}{total\;DNA\;(ng)}\times 100$$

where total DNA = *Fusarium* DNA + maize DNA.

### 4.6. Tissue Preparation for Microscopy Analyses

Entire inoculated or mock maize seedlings were incubated in a mixed solution of chloroform, 96% ethanol, and trichloroacetic acid (1:4:0.15). The fixed roots were subjected to hand sectioning and transferred to a glass slide. At every time point, five primary roots were collected for each genotype, and three parts of each root were sectioned, with three sections from each part. In total, 45 roots sections for each genotype at every time point were observed. All root sections were first emerged in distilled water on a glass slide, and then stained for 5 min by using wheat germ agglutinin Alexa Fluor 488 conjugate (WGA) (Invitrogen, Carlsbad, CA, USA), washed by distilled water three times. Confocal laser scanning microscopy was performed with a Zeiss LSM780 microscope (Carl Zeiss Microscopy Deutschland GmbH, Oberkochen, Germany) after WGA staining. For observation at 510 nm and autofluorescence detection at 550 to 650 nm, a long-pass filter was used.

### 4.7. Metabolomics Analysis Using UPLC-ESI-Q TRAP-MS/MS

Widely targeted metabolite analysis was carried out by the MetWare Company (Metware Biotechnology Co., Ltd., Wuhan, China). Twelve maize seedling root samples were freeze-dried, ground into powder, dissolved in a 70% methanol solution, and then centrifuged for extraction prior to UPLC-MS/MS analysis. The sample extracts were analyzed using an UPLC-ESI-MS/MS system (UPLC, SHIMADZU Nexera X2; MS, Applied Biosystems 6500 Q TRAP). The mass spectrometry data obtained were processed using Analyst software (version 1.6.3). The MetWare database (MWDB) was employed to conduct both qualitative and quantitative analyses of the sample metabolites through mass spectrometry.

### 4.8. RNA Extraction and Gene Expression Analysis

The total RNA of twenty-four samples was extracted using the VeZol Reagent (Vazyme, Nanjing, China). cDNA was synthesized by using the Hifair^®^ III 1st Strand cDNA Synthesis SuperMix (Yeasen Biotechnology Co., Ltd., Shanghai, China). Gene expression analysis was performed using the Roche LightCycler^®^ 480 real-time PCR system (Hoffmann-La Roche Ltd., Basel, Switzerland). For each reaction, 1 μL of template cDNA (1:5) was mixed with 5 μL F488 SYBR mixture (BestEnzymes Biotech Co., Ltd., Lianyungang, China), 3 μL of sterile water, and 1 μL of forward and inverse primer (each 10 pmol/μL). Target gene expression was quantified using the comparative 2^−ΔΔCT^ method [56].

### 4.9. Sample Preparation for Matrix-Assisted Laser Desorption/Ionization Mass Spectrometry Imaging (MALDI MSI)

Stem base tissues were submerged in 4% (wt/vol) CMC solution (carboxymethyl cellulose sodium salt) (MDBio, Inc., Qingdao. China) in a metal mold and fixed by freezing the mold into an isopropanol and dry ice mixture. The CMC block was sliced to 12 μm thickness by using a cryostat (Leica CM1950, Nussloch Germany). The section was then thawed and mounted on a conductive indium–tin oxide (ITO)-coated glass slide. A mixture of CA (20 mg/mL in 50% MeOH), CHCA (30 mg/mL in 80% ACN), HFIP, and TFA (V/V/V/V, 1:1:0.1:0.004) was sprayed on tissue section by using a home-built electric field-assisted matrix coating system.

### 4.10. MALDI MSI and Data Analysis

MALDI MSI was conducted using an ultrafleXtreme MALDI TOF/TOF MS (Bruker Daltonics, Bremen, Germany) fitted with a Nd:YAG solid-state Smartbeam II laser (λ = 355 nm, 2 kHz). The laser was configured to the “Ultra” footprint setting (∼100 μm diameter). Data acquisition was carried out in the m/z range of 100–500 in positive ion reflection mode, with 100 laser shots fired at 1 kHz. Mass spectrometer calibration was achieved using CHCA matrix ions, CA-derivatized theanine, and a Peptide Calibration Standard Kit II (Bruker Daltonics, USA). Putative identification of target ions was performed using a high-resolution 7T solariX Fourier transform ion cyclotron resonance (FTICR) mass spectrometer equipped with a dual MALDI and ESI source (Bruker Daltonics). MALDI MSI data were processed and exported using flexAnalysis 3.4 and flexImaging 4.1 (Bruker Daltonics).

### 4.11. Statistical Analyses

The statistical analysis for metabolomics was conducted using MetaboAnalyst 5.0 online software (www.metaboanalyst.ca, accessed from 1 January 2024 to 31 December 2024) and Metware cloud, a free online platform for data analysis (https://cloud.metware.cn, accessed from 1 January 2024 to 31 December 2024). The statistical analyses for individual metabolites and genes were performed using GraphPad Prism 8.2.1 (GraphPad Software Inc., San Diego, CA, USA) and R package 4.4.2.

## Data Availability

The data that support the findings of this study are available from the corresponding author upon reasonable request.

## Supplementary Materials

The following supporting information can be downloaded at: https://www.mdpi.com/article/10.3390/plants14091376/s1, Figure S1: GRR phenotypes of W438 (resistant) and 335M (susceptible) upon *F. graminearum* infection at 4 dai; Figure S2: Principal Component Analysis (PCA) of metabolites and differentially expressed metabolites (DEM) in W438 and 335M; Figure S3: Dynamic changes in metabolite contents in glycerophospholipid pathway in W438 and 335M at 4 dai; Table S1: Relative fungal biomass of W438 and 335M in three biological replicates and statistical analysis; Table S2: Specific primers for gene expression and fungal biomass assay.

## Abbreviations

The following abbreviations are used in this manuscript:

GRR	Gibberella root rot
PE	phosphatidylethanolamine
PC	phosphatidylcholine
lysoPE	lysophosphatidylethanolamine
lysoPC	lysophosphatidylcholine
PLA	phospholipase
LPLA	lysophospholipase
MSI	mass spectrometry imaging
