# Supplementary material for: The Involvement of Glycerophospholipids in Susceptibility of Maize to Gibberella Root Rot Revealed by Comparative Metabolomics and Mass Spectrometry Imaging Joint Analysis
Source: Plants (Basel). 2025 May 1;14(9):1376. doi: 10.3390/plants14091376 (PMC12073750; doi:10.3390/plants14091376)
Supplement: Supplementary file 1 [file plants-14-01376-s001.zip › supplementary figure.pdf]

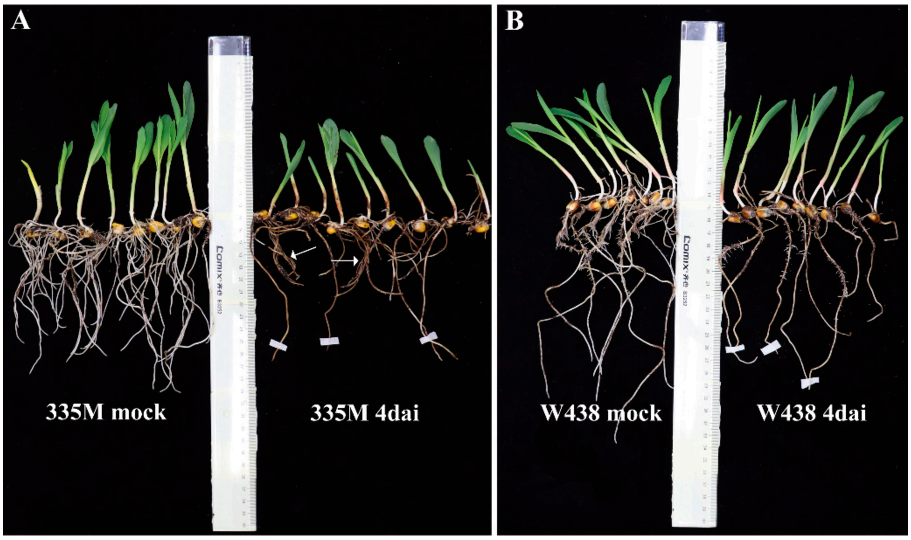

Figure S1. GRR phenotypes of W438 (resistant) and 335M (susceptible) upon *F. graminearum* infection at 4 dai. (A) Growth suppression of root and shoot was observed in 335M at 4 dai. The white arrows indicate severe necrosis on roots. (B) W438 displayed fewer lesion on the root and minimal effect on the shoot growth at 4 dai.

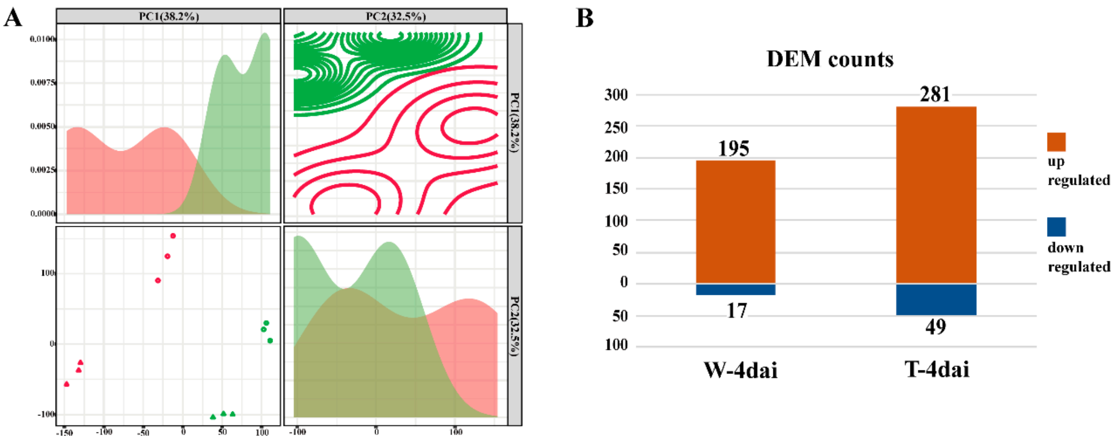

Figure S2. Principal Component Analysis (PCA) analysis of metabolites and differentially expressed metabolites (DEM) in W438 and 335M. (A) PCA analysis exhibited the metabolomics difference between W438 and 335M both before and after infection. (B) In 335M, there were more differentially expressed metabolites. T: 335M, W: W438.

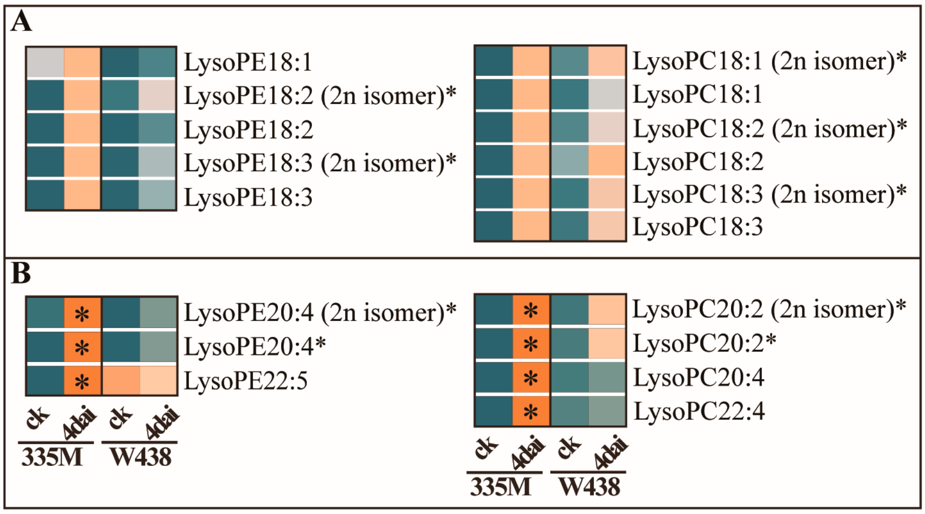

Figure S3. The dynamic changes of metabolite contents in glycerophospholipid pathway in W438 and 335M at 4 dai. (A) The content comparison of lysoPCs and lysoPEs with fatty acid 18:1, 18:2 and 18:3 between W438 and 335M at 4 dai. There were no significant difference between two genotypes. (B) The content comparison of lysoPCs and lysoPEs with long fatty acid 20:2, 20:4, 22:4 and 22:5 between W438 and 335M at 4 dai. Asterisks indicate statistical significance (*$p \leq 0.05$; two-way repeated ANOVA).
